# Hospital Stamps and Out-Patients

**Published:** 1899-02-18

**Authors:** 


### The Institutional Workshop.

### HOSPITAL STAMPS AND OUT-PATIENTS.

### A CHAT WITH THE SECRETARY OF THE CENTRAL LONDON THROAT AND EAR HOSPITAL

It being more than six months since the Secretary of the Central London Throat and Ear Hospital. in Gray's Inn Road, commenced to take the stamps issued by the Prince of Wales's Hospital Fund, I called the other day on Mr. Richard Kershaw, secretary of the hospital since 1878, and the first to take up this matter, for the purpose of ascertaining what sort of progress he had made.

First, I asked him what quantity of Hospital stamps he had taken.

"I took two hundred half-crown stamps in June last year," he replied.

"Had you any difficulty in selling them?"

"None whatever. I think I sold them all by August to the out-patients. I did not get any more until November, owing to the new issue not being available. But since then, and especially during January, they have been sold just as easily. In fact, some of the patients who have come to me to express their gratitude at the treatment received have asked to be supplied with a stamp."

"With regard to the two hundred disposed of, have you applied to the purchasers subsequently?"

"At Christmas I sent 200 circulars to those patients who had bought stamps, and 200 more to those who had been in-patients. The result of the two appeals was the receipt of contributions amounting to £50. I think the very fact of the former patients knowing that by taking a stamp they are helping the hospital may have prompted them to send a donation."

"Of course, the contributions varied in amount?"

"They ran from a shilling to £3, the latter amount being collected by a former in-patient among his friends. The letters expressing thankfulness for benefits received were not less welcome than the contribution."

"I infer that you have not taken any of the shilling stamps?"

"I have not. The system here adopted is that all out-patients are invited to contribute towards their treatment, and two-thirds of them do; but as the average amount of such contribution is only sixpence an attendance, it seems superfluous to ask them to purchase a shilling stamp. Among the patients, however, some who volunteer payment seem in a position to pay more, though still suitable for hospital relief. I have, as far as possible, brought the Prince of Wales's Fund prominently before them, and I have sold some of them a half-crown stamp."

"Would it not be easier to obtain a shilling from them?"

"It might be. But so far as the half-crown stamps are concerned it would obviously complicate accounts if patients paid a shilling, leaving the balance to be paid at a subsequent visit which might not take place. If, however, I asked for a shilling at all, it would be better to ask for it without any question of the 'stamp,' than for a shilling to be paid on account of a half-crown stamp, the balance of which might in some cases never be discharged."

"When I first commenced selling the stamps," continued Mr. Kershaw, "I devoted a great deal of personal attention to the task of interesting the patients in the objects of the Prince of Wales's Fund, and especially in showing how they might aid, not only this hospital, but other hospitals, by purchasing one of the stamps. I found, however, that to do this to twenty or thirty patients in one afternoon involved a demand on my time which I was not justified in supplying."

"But have you no assistance?"

"No, I am single-handed here; and I have come to the conclusion that it would not pay the hospital to employ a clerk for this special work. Moreover, if the patients are to be thoroughly instructed, the question must be brought before them in an interesting manner. I have done this as far as I can with, I think, advantage to the Prince's Fund; but it is impracticable to put the matter before the whole of the out-patients."

"And the in-patients?"

"These are admitted in first by payment—this averages 7s. a week; the second, and the majority, who are unable to pay, are admitted on the urgency of their case. I could not ask the in-patients to buy stamps, but all are told about the Fund, and some of them have taken stamps."

"Have you had any of the five and ten shilling stamps?"

"Yes, I took some, and so far I am sorry to say I have not sold one to a patient. This is another evidence that they have not the money to buy them. Therefore, so long as I have the privilege of selling the stamps I shall keep to the half-crown issue. It is really wasting time with a patient to ask him to buy a shilling stamp when he can only give a shilling as his total contribution."

"How about the albums?"

"I have sold a hundred of them, but they are not selling now."

"Perhaps you have thought how the system might be applied in other hospitals?"

"Yes, I have thought a great deal, and I feel convinced, from my own experience here, where the payment system is already in vogue, that it could be adapted easily to any general hospital in London."

"What is the basis of your confidence?"

"Assuming that the class of patients coming to this hospital afford a fair sample of those admitted at any other, the fact that over fifty per cent. of them willingly give a contribution of sixpence 'an attendance' is a strong basis for the conviction that a large number of stamps should be sold at a general hospital where, with one or two exceptions, patients are not asked to make a contribution. The out-patients here contribute over £1,000 a-year."

"But might it be urged that your out-patients are better able to pay than the out-patients at a general hospital?"

"Twenty years ago, Sir Henry Burdett, after having made it his business to compare some of the applicants for relief at special hospitals with those at general hospitals, said, 'There is practically no difference as to the class. The hospital patient of London is pretty much the same, if you average him, whether he is in a special department of a general hospital, or in a special hospital, or is an ordinary out-patient of a general hospital.' My own opinion is precisely the same, and I am satisfied that there is absolutely no difference between the out-patients who come here and the out-patients who attend the Royal Free Hospital in this street. Our out-patients render their department self-supporting, and there is not a general hospital in London which could not do the same, either by inviting a voluntary payment from patients or by bringing before their notice, as I have done, the stamps."

"May not their constitution or rules prevent them from exacting a direct payment?"

"Possibly they may. But there can be no such difficulty in regard to the stamps. If a patient is asked to pay for medicine or surgical dressings, why may he not be invited to buy a stamp?"

"Why do your patients look a little more prosperous than those who go to the Free Hospital?"

"Because (1) no less than one-fourth of them are patients sent by their family doctors to obtain expert opinion for which they are unable to pay full fees, and a further large proportion are of the artisan class who, possessed of a desire to contribute, however inadequately, to their treatment, have also sufficient self-respect to look as clean as they can; (2) the remainder copy their example, and, although poor, try to look respectable. During the 21 years I have been here I have never had a disagreeable incident with a patient about the payment question. Our rule is so well known that patients invariably ask, 'What shall I contribute?' or 'I should like to give something.' I think that, when once the system whereby money is raised through the stamps is fully understood, there will be no difficulty in selling them."

"Now, finally," I said, as I watched some of the out-patients in the dispensary hearing and answering questions, "have the stamps been of any direct value to the Central Throat and Ear Hospital, or not?"

"Undoubtedly they have. We are, so far, the richer by £25 in hard cash, which is derived from this branch of the Prince of Wales's Fund, with the fruits of increased interest in our work by the stamp purchasers."

